# Interactive effects of dopamine transporter genotype and aging on resting-state functional networks

**DOI:** 10.1371/journal.pone.0215849

**Published:** 2019-05-08

**Authors:** Christian Baeuchl, Hsiang-Yu Chen, Yu-Shiang Su, Dorothea Hämmerer, Manousos A. Klados, Shu-Chen Li

**Affiliations:** 1 Faculty of Psychology, Technische Universität Dresden, Dresden, Germany; 2 Graduate Institute of Brain and Mind Sciences, College of Medicine, National Taiwan University, Taipei, Taiwan; 3 Taiwan International Graduate Program in Interdisciplinary Neuroscience, National Taiwan University and Academia Sinica, Taipei, Taiwan; 4 Institute of Cognitive Neuroscience, University College London, London, United Kingdom; 5 Institute for Cognitive Neurology and Neurodegenerative Diseases, Otto-von-Guericke Universitaet, Magdeburg, Germany; 6 German Center for Neurodegenerative Diseases, Magdeburg, Germany; 7 Department of Biomedical Engineering, School of Life and Health Sciences, Aston University, Birmingham, United Kingdom; University of Udine, ITALY

## Abstract

Aging and dopamine modulation have both been independently shown to influence the functional connectivity of brain networks during rest. Dopamine modulation is known to decline during the course of aging. Previous evidence also shows that the dopamine transporter gene (*DAT1*) influences the re-uptake of dopamine and the anyA9 genotype of this gene is associated with higher striatal dopamine signaling. Expanding these two lines of prior research, we investigated potential interactive effects between aging and individual variations in the *DAT1* gene on the modular organization of brain acvitiy during rest. The graph-theoretic metrics of modularity, betweenness centrality and participation coefficient were assessed in 41 younger (age 20–30 years) and 37 older (age 60–75 years) adults. Age differences were only observed in the participation coefficient in carriers of the anyA9 genotype of the *DAT1* gene and this effect was most prominently observed in the default mode network. Furthermore, we found that individual differences in the values of the participation coefficient correlated with individual differences in fluid intelligence and a measure of executive control in the anyA9 carriers. The correlation between participation coefficient and fluid intelligence was mainly shared with age-related differences, whereas the correlation with executive control was independent of age. These findings suggest that *DAT1* genotype moderates age differences in the functional integration of brain networks as well as the relation between network characteristics and cognitive abilities.

## Introduction

During alert but task-free states, the brain’s sensorimotor and higher cognitive systems display organized temporal correlations between spontaneous fluctuations of brain activity in different brain regions. Over the past decade, functional magnetic resonance imaging (fMRI) research on the organized patterns of spontaneous brain activity suggests that resting-state functional connectivity (rsFC) is a promising avenue for investigating the dynamics of coherent brain activity patterns that are organized into distinct systems or networks (for a recent review see [1]). The rsFC can be calculated using different methodologies, including: a seed-based approach by selecting voxels in the regions of interests (ROI) as seeds for cross correlational analyses (e.g., [2], [3]), independent component analysis (ICA; [4]), as well as the more recent clustering methods (e.g., [5]). A multitude of factors have been shown to be associated with interindividual differences in the patterns of rsFC, notably: brain maturation (for a review see [6]), brain aging (for review see [7]), neuromodulation (e.g., [8]), and clinical conditions (for reviews see [9], [10]).

Of particular relevance here are changes in rsFC during the course of aging. A range of previous studies have investigated the effects of aging on brain functional connectivity by measuring age differences within networks using seed-based or ICA analyses. So far the most consistent finding is the observation of reduced intranetwork functional connectivity among regions in the default mode network (DMN) in older compared to younger adults (e.g., [11]; see [7], [12] for overviews). The DMN is a collection of brain regions, including posterior cingulate cortex, precuneus, medial prefrontal cortex and the lateral parietal cortex [13], that are known to implicate high-level cognitive processes, such as episodic memory and self-referential processing [10] and that are sensitive to age-related neurodegenerative processes [10, 14]. Of note, using a probabilistic ICA method Damoseaux et al. [11] showed reduced rsFC in the DMN in older relative to younger adults and the strength of rsFC in the anterior DMN being negatively correlated with age in older adults. Furthermore, age-related decreases in intranetwork rsFC in the dorsal (DOR), ventral/saliency (VEN) attention, or somatomotor (SOM) network have also been observed in several studies (e.g., [11], [15]). Of note, Betzel et al. [15] investigated age-related differences both in intranetwork and internetwork rsFC in a lifespan sample and found opposite results: while intranetwork connectivity decreased with age, internetwork connectivity increased. Similarly, in other cross-sectional studies of internetwork connectivity between the dorsal attentional network, DMN, and the frontoparietal control network has been found to be increased in older compared to younger adults (e.g., [15], [16], [17]), suggesting attenuated system segregation in old age [18]. However, other studies have reported aging-related longitudinal decline of internetwork functional connectivity between the executive control network and the DMN in the young-old age range (60 to 75 yrs; see [19]), reduced internetwork connectivity between the DMN and dorsal attention network in childhood as well as in old age [20], or no age differences in the connectivity between the DMN and the frontoparietal control network [21]. Thus, unlike the evidence for aging-related decrease in intranetwork rsFC, findings about effects of age on internetwork rsFC are less consistent.

Other than aging, neuromodulation has also been shown to affect rsFC. Since neurotransmitter systems (e.g., the monoamines) play a prominent role in affecting brain functions [22, 23], the question of whether rsFC may be modulated by the efficacy of neurotransmitter systems has also been explored. Of particular relevance here are initial findings from pharmacological, genotype and patient studies, which suggest that dopamine modulates the rsFC. Regarding pharmacological investigations in healthy samples, using the seed-based approach, a recent study by Farr et al. [24]showed that methylphenidate (MPH), a blocker of dopamine and norepinephrine transporters, altered rsFC between the dorsal striatum, the motor cortex, the hippocampal memory circuit and the prefrontal cortex in younger adults. Specifically, whereas a single dose of MPH (45 mg) increased connectivity between striatal regions and the motor cortex as well as the hippocampal memory circuit, connectivity within the prefrontal regions was reduced. By blocking dopamine and norepinephrine receptors, MPH increases synaptic transmitter levels. The attenuation of striatal-frontal connectivity might result from MPH shifting frontal dopamine/norepinephrine beyond the optimal level. As for studies in clinical populations, another study by Yang et al. [25]explored the effects of levodopa on rsFC in Parkinson’s (PD) patients, who characteristically suffer from nigrostriatal dopamine loss. The results showed that, during the OFF medication state, the rsFC in striatal seed regions (dorsal caudate, ventral putamen and dorsal putamen) was lower in PD patients compared to healthy age-matched controls. Furthermore, dopamine medication had differential effects on rsFC in PD patients: levodopa reduced the functional connectivity between ventral striatal seeds with the ventral medial, dorsal frontal and orbitofrontal regions, whereas the connectivity between the dorsal striatal seeds with the primary and secondary motor regions was increased. Relatedly, the regional rsFC between the midbrain and the putamen in PD patients and demented patients with Lewy bodies had been shown to correlate positively with the availability of striatal dopamine transporters [26], suggesting that individual differences in the availability of dopamine transporters may moderate the effects of dopamine pharmacology on rsFC. These initial findings notwithstanding, thus far pharmacological studies on this topic are still scarce and the currently available results are too heterogeneous to determine systematic effects of dopamine modulation on rsFC. In this context, individual variations in dopamine genes, which could moderate pharmacological effects, need to be considered as well.

Indeed, another line of research explored the effects of individual differences in the variations of dopamine transporter gene on rsFC [8, 27]. Of note, the human dopamine transporter gene (*DAT1*) displays a polymorphic 40-base pair (bp) variable number of tandem repeats (VNTR). The 40-bp VNTR element is repeated between 3 and 13 times, with the highest frequency in the 9-repeat and 10-repeat form [28]. Variations in the *DAT1* VNTR polymorphism have been related to the efficacy of dopamine reuptake from the synaptic cleft back to the presynaptic terminals. The 10-repeat homozygotes are associated with higher striatal dopamine transporter density than the 9-repeat homozygotes, resulting in lower levels of extrasynaptic dopamine in 10-repeat homozygotes [29, 30]. In a study of younger adults by Gordon et al. [8], dorsal caudate seeded rsFC with the insula, dorsal anterior cingulate cortex, and the dorsal lateral prefrontal cortex have been found to be stronger in the A9 homozygotes and A9/A10 carriers (i.e., in anyA9 carriers) than in the A10 homozygotes of the *DAT1* gene.

Taken together, to date studies about dopamine genotype effects on rsFC are still scarce and the effects of dopamine pharmacological interventions are rather differential, which is, in part, due to the non-linear dose-response relationship of dopamine signaling [31] as well as individual differences in genotype and/or age. During the course of aging, various facets of the dopamine systems (e.g., availabilities of receptors and transporters) decline gradually and substantially (see [32] for review). Of particular relevance here is a clear age-related decline in dopamine transporter binding in striatal regions (caudate and putamen) from early to late adulthood (e.g. [33, 34]). Age-related loss of dopamine transporter has been found to be associated with aging-related memory deficits (e.g. [33, 35]). Furthermore, variations in the *DAT1* VNTR polymorphism were found to interact with other dopamine genes and were associated with individual differences in sequence learning [36] and serial memory [37]. Compared to carriers of the A10 allele, older *DAT1* A9 homozygotes learned more about the sequential structure during sequence learning [36] and recalled more correctly during serial recall [37]. Taken together, although the aforementioned studies have shown that aging and genetic variations independently impact rsFC, thus far how aging, which is associated with reduced dopamine transporter variability, might interact with dopamine transporter genotype in influencing spontaneous brain functional connectivity has not yet been explored. Thus, the main aim of this study is to investigate potential interactive effects of aging and the *DAT1* genotype on rsFC in an adult age comparative sample by taking a graph theoretical approach.

In studies involving multiple cohorts as in the case of age comparative samples, the applicability of seed-based and independent component analyses are rather limited, because age-related differences in the regions of interests at the structural and functional level as well as in the numbers of extracted independent components render it difficult to directly compare groups [38]. In recent years, graph theory based approaches have been fruitfully applied to analyze and characterize structural and functional brain networks. Graph theory provides a wide range of metrics for characterizing different properties of networks, such as their tendency to form modules (modularity), within and between-module connectivity, characteristic path length, local and global efficiency, as well as hub architecture (see [1] for review). These measures can be used to quantify individual differences in brain functional connectivity that are related to age, genetic variations, clinical conditions or their interactions. For instance, Brier et al. [39] employed graph theoretical analyses to study age- and dementia-related differences in rsFC and found an age-related decrease in modularity. Furthermore, participation coefficient, which quantifies how evenly distributed a node’s connections are across a set of modules (see [1]), was associated with a clinical dementia rating, with lower participation coefficient being associated with greater dementia severity. Extending the prior studies reviewed above, in this study we investigate potential effects of *DAT1* genotype on adult age differences in rsFC in healthy younger and older adults. Given consistent findings of age-related decrease in intranetwork connectivity (e.g., [11], [15]), it is expected that the graph-theoretical metrics characterizing the modular architecture of rsFC would be altered in aging brains and such effects would interact with *DAT1* genotype.

## Methods

### Subjects

The study sample consists of 41 younger adults (17 male, age range: 20–33 years, mean age: 26.44 ± 3.15 SD) and 37 older adults (21 male, age range: 60–73 years, mean age: 66.38 ± 3.43 SD). Subjects were recruited from the participant pools of the Center for Lifespan Psychology, Max Planck Institute for Human Development in Berlin, Germany (for a previous report on structural brain measures of this dataset, see FitzGerald et al. [40]. All subjects were right-handed (as assessed by the Oldfield Questionnaire [41]: LQ > 80). None of the subjects reported cardiovascular pathology, psychotropic medication usage, history of neurological or psychiatric episodes or substance abuse. A further exclusion criterion was the current intake of medication that could interact with the dopamine system. In addition, subjects were instructed not to drink alcohol, coffee or smoke prior to the experiment. Ethic approval in accordance with the Helsinki declaration was granted by the ethics committee of the Charité, University Medicine Berlin. Participants provided written informed consent prior to study participation and were paid € 10 per hour of the experiment.

### Cognitive measures

Prior to the imaging session, measures of basic cognitive abilities were assessed in a behavioral assessment session. Of relevance here, the identical-pictures test [42] and a German variant of the spot-a-word test [43] for characterizing the sample characteristics were assessed. As expected, performance on the identical-pictures test was lower in older than in younger adults (t_(71)_ = 9.03, p < 0.001), whereas performance on the spot-a-word test was higher in older than in younger adults (t_(72)_ = -2.58, p < 0.05). This observed dissociation between adult age gradients of basic processing speed and verbal ability in our sample is consistent with data from population-based lifespan samples [44]. Furthermore, general cognitive ability was assessed by the Raven’s test [45] and executive control function was assessed by a variant of the Stroop task [46].

### Genotyping

Saliva samples were taken from subjects using the Oragene DNA sample collection kit (ON, Canada). DNA was extracted from these saliva samples using standard techniques. TaqMan probes for the genotyping were designed and synthesized by Applied Biosystems (Foster City, CA, USA). We selected the variable number of tandem repeats (VNTR) polymorphism of the dopamine transporter gene *DAT1* (also known as *SLC6A3*) based on previous studies showing associations between these polymorphisms of the *DAT1* gene and functional brain connectivity during task and rest in younger adults [8, 27]. We restricted the sample to include 9-repeat (A9) and 10-repeat (A10) allele carriers only. As the frequency of the A9/A9 group is very low, we compared two *DAT1* genotype groups: carriers with at least one A9 allele (i.e, the anyA9 genotype, which included A9/A9 and A9/A10; n = 41) and A10 homozygotes (A10/A10; n = 37). The number of participants with these two genotypes seprated by age groups are as follows: n = 23 (YA) and n = 18 (OA) for anyA9 and n = 18 (YA) and n = 19 (OA) for A10 homozygotes. The observed allele frequencies in our sample did not deviate significantly from the Hardy-Weinberg equilibrium (χ^2^ < 0.05; p > 0.05).

### Imaging protocols

Magnetic resonance imaging (MRI) was performed using a 3 tesla Siemens Trio Tim whole body scanner (Erlangen, Germany) at the Charité Benjamin Franklin Campus. Resting-state functional MRI (rsfMRI) data were obtained using a T2*-weighted echo-planar imaging sequence (TR = 2500, TE = 30 ms, FA = 80°, FOV = 1296 x 1296 mm, aquisition matrix: 72 x 72, voxel dimensions: 3 x 3 x 3 mm). 36 slices were acquired in an interleaved descending order for whole brain coverage. A run of resting-state imaging resulted in 180 volumes and took 7.5 minutes. In addition, a high-resolution 3D T1-weigthed magnetization-prepared rapid gradient-echo (MPRAGE) image (TR = 1550 ms, TE = 2.34 ms, FA = 9°, FOV = 244 x 244 mm, acquisition matrix: 256 x 256, voxel dimensions = 1 x 1 x 1 mm) was acquired for normalization to template space.

### Preprocessing of resting-state fMRI

Preprocessing of resting-state fMRI data was carried out using SPM 12 (http://www.fil.ion.ucl.ac.uk/spm/; Wellcome Trust Centre for Human Neuroimaging, UK) and FSL (https://fsl.fmrib.ox.ac.uk/fsldownloads_registration; FMRIB, Oxford, UK). All functional data were slice-time corrected using SPM's Fourier phase shifting interpolation, followed by head motion correction with unwarping based on the gradient field map. The T1-weighted image was coregistered to the mean functional image and segmented into grey matter and white matter images. The segmented images were used to normalize the functional images to the standard space of the Montreal Neurological Institute (ICBM 152 MNI template) via SPM’s DARTEL toolbox. Functional images were resampled to the original acquisition resolution of 3 mm cubic voxels and spatially smoothed (8 mm full width at half maximum Gaussian kernel). Additional head-motion correction was performed using FSL's ICA-AROMA, a data-driven method to identify and remove motion-related independent components from the fMRI data. The preprocessed functional data were then detrended and subjected to a bandpass filter which attenuated signal below 0.009 Hz and above 0.08 Hz in voxel timecourses. Voxelwise regression was used to remove the effects of the mean white matter signal and the mean cerebrospinal fluid signal from the data. Since head motion can have particularly detrimental effects on functional connectivity estimations by introducing spurious correlations between timecourses of rsfMRI data [47], an additional voxelwise outlier censoring procedure as described by Aurich et al. [48] was applied to the data. Outlier time points are based on the signal intensity of individual voxels across the time series. To this end the median m(v) and the median absolute deviation MAD(v) are calculated for each voxel. Then an intensity range for each voxel is defined by [m(v)—a * MAD(v); m(v) + a * MAD(v)], where a = Q^-1^(0.01/N) * (π/2)^1/2^ and Q is the inverse Gaussian cumulative distribution function and N is the length of the time series. Voxels outside this intensity range are considered outliers. The total amount of outlier voxels in the brain is calculated for each time point. If a given time point is marked as an outlier in at least 10 percent of voxels, then this time point is removed for all the voxels in the brain for that subject. Over all subjects, the total percentage of voxels removed from the data by this procedure is 0.03 percent.

### Node definition and functional connectivity graph

**Fig 1:**
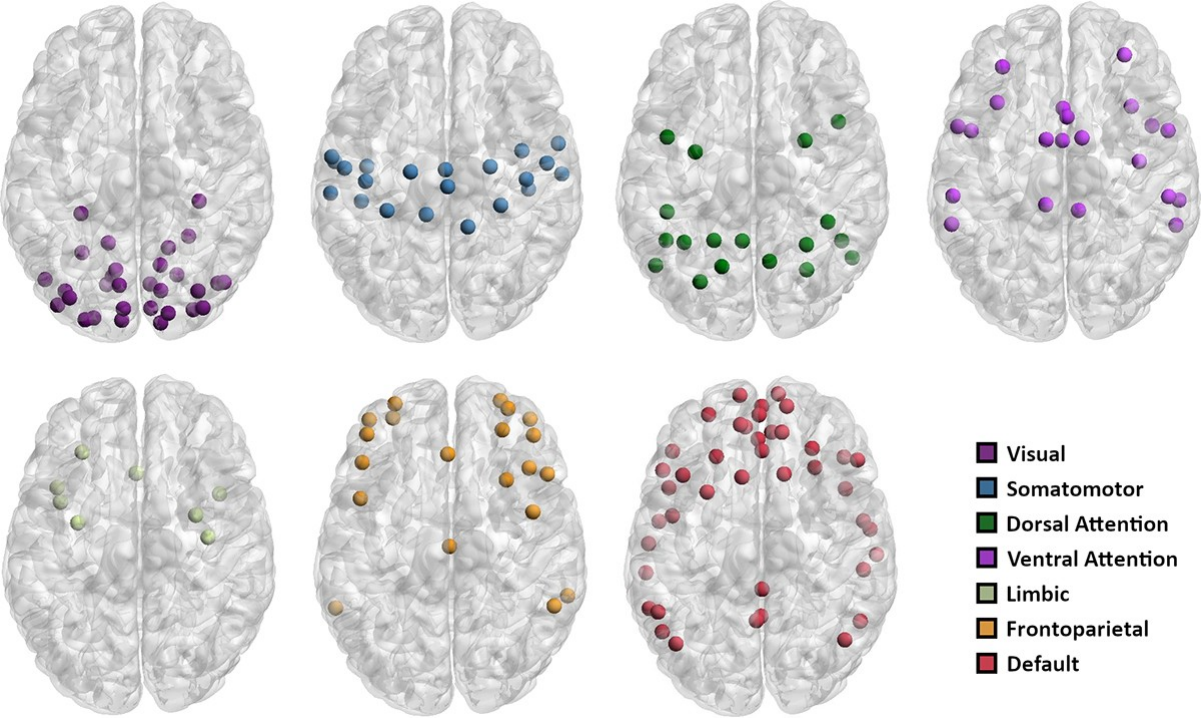
Cortical networks. Maps with the a-priori assignment of the 200 nodes to partitions of seven networks (from Yeo et al. [50]).

The calculation of (functional) connectivity networks (i.e. “graphs”) requires the definition of network nodes. In large-scale brain networks, these nodes typically represent non-overlapping portions of functionally or anatomically defined brain regions. We used a publicly available whole-brain atlas that is subdivided into 200 regions (http://ccraddock.github.io/cluster_roi/atlases.html). These regions of interest (ROI) were generated using a data-driven method which subdivides resting-state fMRI data into clusters based on functional similarity of voxels [49]. The method allows a varying number of ROIs, whereby a 200-ROI parcellation scheme keeps the balance between anatomic variability (too few ROIs would combine anatomically distinct regions) and interpretability (too many ROIs might produce neighboring clusters with low distinctiveness). Nodes were assigned to one of seven large-scale cortical networks defined by Yeo et al. [50] (see Fig 1), which are calculated based on intrinsic functional connectivity. The purpose of this assignment was to investigate network specific effects of graph-theoretic metrics alongside examinations of whole-brain networks. Functional connectivity graphs were estimated by computing pairwise zero-lag Pearson correlations between the average time series of each of the 200 ROIs with the average time series of all other ROIs, excluding autocorrelations. The resulting correlation coefficients were then transformed into z scores using the Fisher z(r) transformation in order to normalize their distribution. In this paper we focus only on the positive correlations, while the negative ones were set to 0. Functional networks can either be weighted (the strength of connections is retained) or unweighted (a binary or “adjacency” matrix in which n_ij_ = 1 denotes a connection between nodes i and j, whereas n_ij_ = 0 indicates the absence of such a connection) [51]. We opted for unweighted graphs as they can be more easily interpreted [52].

After obtaining the correlation matrices for each of the participants, we used a binary graph method similar to Brier et al. [39] to obtain an unweighted graph for each of the subjects. Specifically, the corresponding correlation matrices were thresholded such that correlation coefficients exceeding the threshold were set to 1, while those values that did not reach threshold were set to 0. Rather than setting a fixed threshold (T) and applying it to the correlation coefficients, we varied T over a range of values. The values for T were chosen such that every subject’s network had identical average degree centrality (K). Degree centrality is a nodal parameter that is defined as the the number of edges (E) that are connected to a specific node, while K is the average degree centrality across all nodes. In order to create networks that have a specific K, proportional thresholding was used (see [39] for further details). Specifically, the range of K was set from 6 to 90, meaning that the number of edges (E) varied from 3–45% of a fully connected graph with 200 nodes.

### Grapth-theoretic metrics

All graph-theoretic metrics measures were computed using the Brain Connectivity Toolbox ([53], https://sites.google.com/site/bctnet/). In light of results from the prior study by Brier et al. [39], we focused specifically on 3 metrics: modularity (Q), participation coefficient (PC), and betweenness centrality (BC).

#### Modularity

The degree to which a network structure can be decomposed into separate nonoverlapping subsets of nodes is expressed in a single statistic called modularity (Q) and computed by the following equation:

$$Q=\underset{i\in M}{\sum }[C_{ij}-(\underset{j\in M}{\sum }C_{ij}{)}^{2}]$$

where *i* and *j* are different modules in the set of all modules *M*, and *C* is the proportion of existing connections between two modules. Nodes within a particular module are more densely connected to each other than to nodes outside that module. Consequently, computing modules requires finding the partition that maximizes the ratio between within-group edges and between-group edges [51, 53]. Several algorithms exist that use initially an arbitrary module structure and optimize it using an iteration procedure. Some of them allow hierarchical modules, where smaller node communities can be nested in larger ones [54] while others are able to detect overlapping community structures, where nodes can be members of several different modules at once [55]. In this study we focused on the subdivision of graphs into nonoverlapping, unweighted and undirected modules (cf. [39]). For computing modularity, we initialized the modules to be identical to the seven cortical networks by Yeo et al. [50] (see Fig 1), while the algorithm re-assigns the nodes to different modules depending on the edge distribution. Hence, it functions as a default starting decomposition that can be rejected in cases where the graph edges don’t support this modular decomposition.

#### Participation coefficient

After a graph has been partitioned into modules, the nodes of a module can be characterized by how they are connected within the module as well as with other modules. To this end, the participation coefficient (PC) compares the number of links of a node *i* to other nodes in the set of modules *s* $\left(\kappa_{s_{i}}\right)$ with it's number of links within its own cluster (*κ*_*i*_):

$$P_{i}=1-\overset{n_{M}}{\underset{s=1}{\sum }}(\frac{\kappa_{s_{i}}}{\kappa_{i}}{)}^{2}$$

If the value of PC is close to 1, the connections distribute evenly across modules, whereas if the value is close to zero, most connections are only within its own module. The intermediate values of PC can be used to heuristically characterize the role of a node as peripheral, connector or kinless [56]. In our analyses, the PC values were averaged either across the whole brain or within each of the seven YEO networks to obtain global and subnetwork-based measures.

#### Betweenness centrality

Several metrics of centrality exist which assess the importance of a node for functional integration within a network. Nodes with high centrality interact with many other nodes within a network and therefore potentially function as hubs for the given network. Betweenness centrality (BC) is an important measure in this regard since it captures the degree to which a node lies on the shortest path between any two given nodes. A node with high values of BC participate in a large number of shortest path in a network. Thus, a node with high BC potentially controls the flow of information in a network, since a significant amount of information passes through it [57]. For any given node *v* BC is defined as follows:

$$BC(v)=\underset{s,t:s\neq v\neq t}{\sum }\delta_{st}(v)$$

where *δ*_*st*_(*v*) denotes the fraction of the shortest paths between the nodes *s* and *t* that pass through node *v*: $\delta_{st}(v)=\frac{\lambda_{st}(v)}{\lambda_{st}}$. For calculating BC, the functional connectivity correlation matrices were binarized (with a value of 1 for each correlation coefficient > 0) and BC values were averaged as it was done for PC.

### Statstical data analyses

To test for potential interactions between age and genotype on rsFC, subjects were split into groups based on age (YA = young adults, OA = old adults), genotype (anyA9 and A10/A10 allele carriers of the *DAT1* gene) and the factorization of the two between-subject factors. We calculated Q, PC and BC for every subject and statistically tested for differences between groups using one-way and two-way analyses of variance (ANOVA). As these tests are carried out separately for every level of node-degree (with K ranging from 6 to 90), we corrected for multiple comparisons using the false discovery rate (FDR) correction [58] to get an adjusted significance level of p < 0.05. Relations between graph-theoretical metrics with behavioral performance were evaluated using Kendall's tau(b) rank correlations.

## Results

As an overview, results for the metric PC showed significant age x genotype interactive effects after FDR correction and are presented in details with follow-up analyses and corresponding figures below. Results for the other two metrics (Q & BC) did not show any significant effects after FDR correction, and are thus only briefly described below.

### Modularity: Effects of age and genotype

Differing from our hypothesis, at the whole-brain level we found no difference in Q between groups, neither for age (YA vs. OA) nor for genotype (anyA9 vs. A10/A10). None of the p-values survived FDR correction for any level of K (see S1 and S2 Figs for analyses of main and interactive effects, respectively).

### Betweenness centrality: Effects of age and genotype

Similar to results found for Q, at the whole-brain level BC did not differ between groups, neither for age (YA vs. OA) nor for genotype (anyA9 vs. A10/A10). None of the p-values survived FDR correction for any level of K (see S3 and S4 Figs for analyses of main and interactive effects, respectively).

### Participation coefficient: Effects of age and genotype

**Fig 2:**
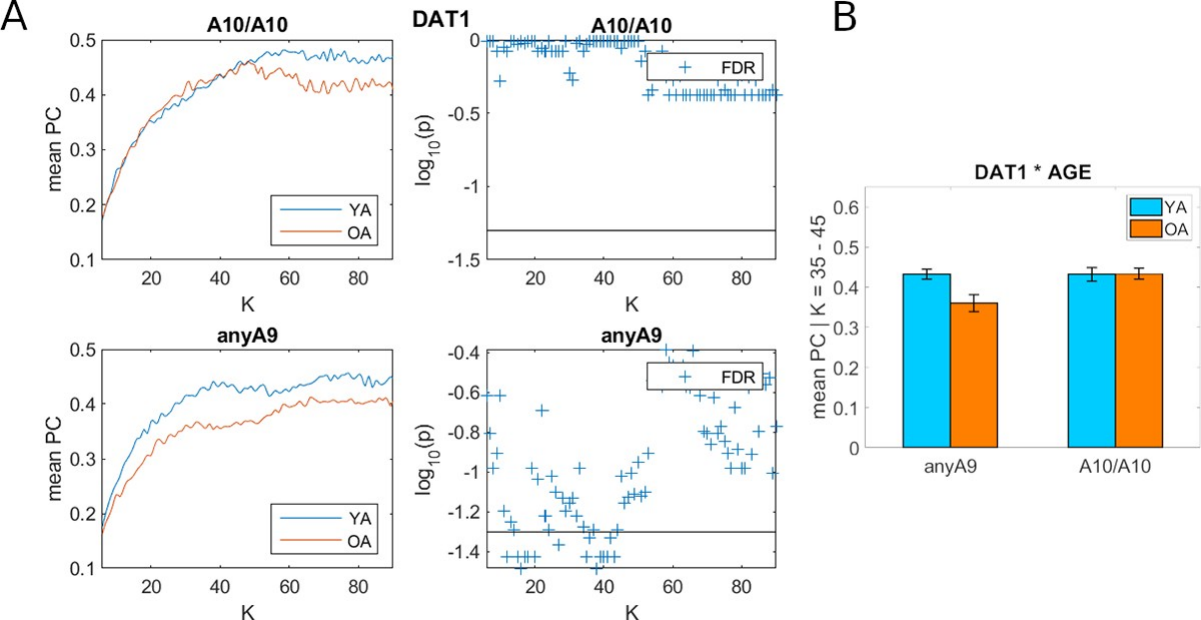
Interactions between age and *DAT1* genotype on participation coefficient (PC): Whole brain. (A) The right panels plot the log of p values as a function of K while the horizontal black line indicates the FDR corrected p = 0.05. (B) Effects shown with averaged PC (over the K range from 35 to 45) as the independent variable and the factors age and gene. Abbreviations: FDR, false discovery rate; K, average node-degree; OA, old adults; YA, young adults.

At the whole-brain level, although younger age or carrying the *DAT1* genotype A10/A10 nominally was associated with higher PC than older age or carrying the genotype anyA9 respectively, these effects did not reach significance after FDR correction across all K levels (see S5 Fig). Crucially, however, the age x genotype interaction yielded significant effects, showing that the age effect on PC was observed in carriers of the *DAT1* anyA9 genotype that is associated with a higher extrasynaptic dopamine level: specifically, higher levels of PC were observed for younger than older anyA9 carriers at the lower K range, whereas such effects were not present in the A10/A10 carriers (Fig 2A). We thus followed up these effects with an additional two-way ANOVA analysis using the averaged PC (over K levels between 35 and 45) as the dependent variable and the factors age group (YA vs. OA) and *DAT1* genotype (anyA9 vs. A10/A10) as between-subject factors. The results showed significant effects (see Fig 2B) for age group (f_(1,74)_ = -4.95, p < 0.05), *DAT1* genotype (f_(1,74)_ = 5.16, p < 0.05) and the age x *DAT1* interaction (f_(1,74)_ = 5.31, p < 0.05).

**Fig 3:**
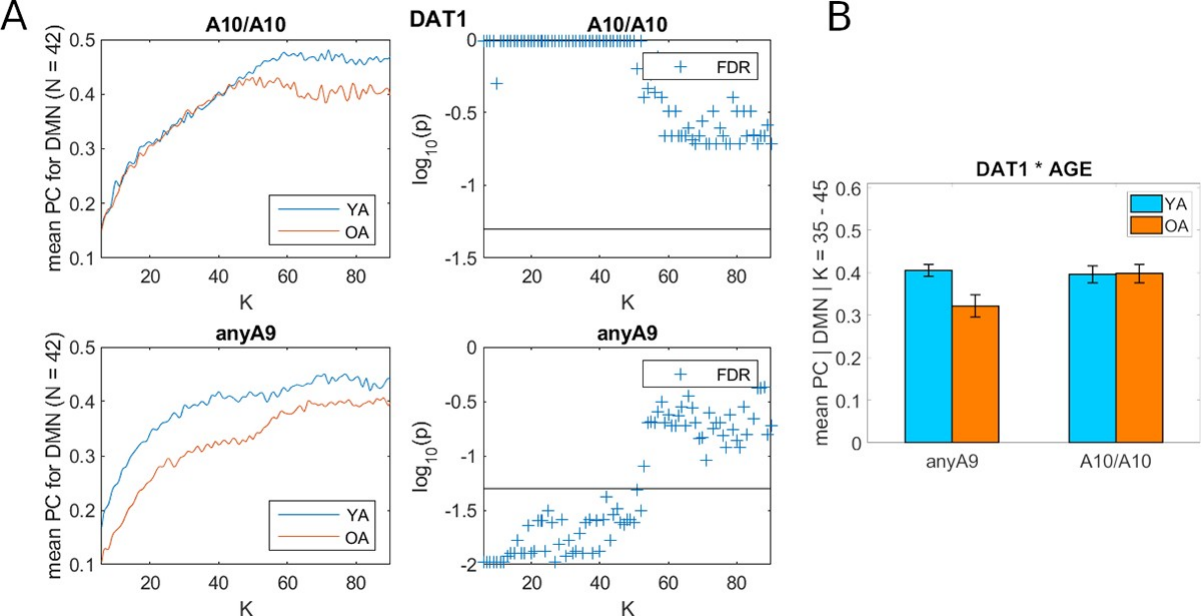
Interactions between age and *DAT1* genotype on participation coefficient (PC): DMN network. (A) The right panels plot the log of p values as a function of K while the horizontal black line indicates the FDR corrected p = 0.05. (B) Effects shown with averaged PC (over the K range from 35 to 45) as the independent variable and the factors age and gene. Abbreviations: DMN, default mode network; FDR, false discovery rate; K, average node-degree; N, number of nodes within subnetwork; OA, old adults; YA, young adults.

In order to explore whether this effect is present throughout the brain or is restricted to certain subnetworks, the same statistical comparisons were carried out on the 7 YEO cortical parcellation networks. Results revealed that the effect of significantly higher PC for younger anyA9 carriers was specific to the default mode network (Fig 3) and was not found for any of the 6 other YEO parcellation networks (S6 Fig). A two-way ANOVA for the DMN network, with PC averaged across the same K range and factors as the whole-brain analysis described above, yielded significant effects of age group (f_(1,74)_ = 4.07, p < 0.05) and the age x *DAT1* genotype interaction (f_(1,74)_ = 4.42, p < 0.05), but not for the factor *DAT1* (Fig 3).

### Relations between participation coefficient and cognition

To explore whether the graph-theoretic metric PC has cognitive relevance, we correlated the averaged PC (over the K range from 35 to 45) with the following cognitive variables: scores on the Raven's matrices test [45] as a measure of general cognitive ability and the performance (accuracy and reaction time) from the incongruent color naming condition of the Stroop task [46] as an indicator of executive control. As anticipated, both cognitive tests revealed age-related differences with higher scores on the Raven's test for younger than for older subjects (t_(73)_ = 8.68, p < 0.001) as well as more accurate (t_(72)_ = 2.50, p < 0.05) and faster responses (t_(72)_ = -10.33, p < 0.001) on the Stroop task in younger compared to older adults.

Correlations were computed separately for all four combinations of age and gene groups Some of the data used for the correlations did not follow a normal distribution, therefore the non-parametric Kendall's tau (b) rank correlation was applied. We opted for Kendall's tau rather than Spearman's rho as the former has been shown to be more robust and efficient than the latter method [59]. FDR corrections were applied as appropriate. None of the calculated correlation coefficients reached significance after correction. This lack of significant findings might be due to the small sample size in each of the four groups.

**Fig 4:**
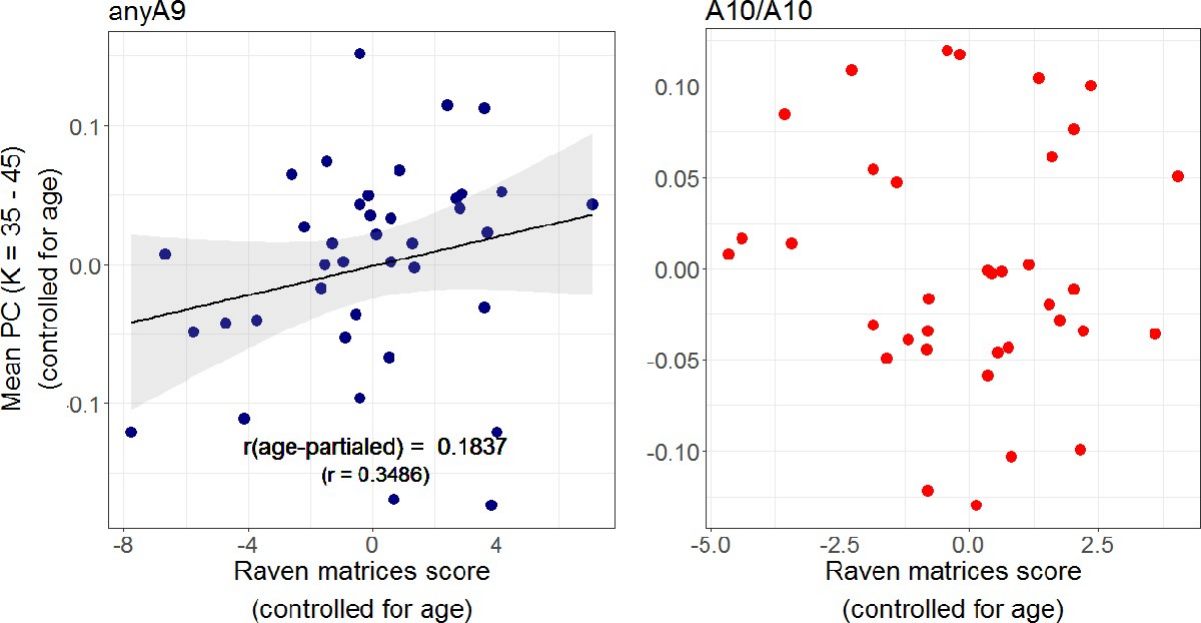
Kendall's tau(b) rank correlations between PC and Raven's matrices scores. Correlations were carried out separately for anyA9 and A10/A10 allele carriers in the entire sample. Effects of age were removed from variables prior to the analyses. The correlation was only significant in the anyA9 group. Abbreviations: K, average node-degree; PC, participation coefficient.

**Fig 5:**
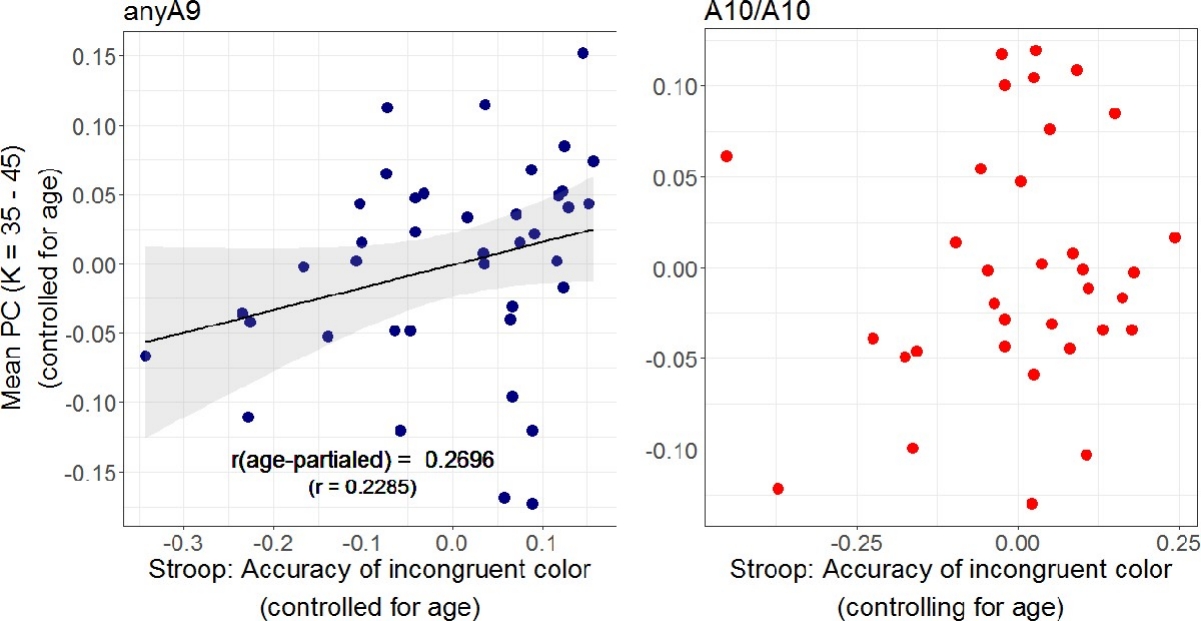
Kendall's tau(b) rank correlations between PC and performance accuracy of Stroop task in the incongruent condition. Correlations were carried out separately for anyA9 and A10/A10 allele carriers in the entire sample. Effects of age were removed from variables prior to the analyses. The correlation was only significant in the anyA9 group. Abbreviations: K, average node-degree; PC, participation coefficient.

Therefore, in the next step, we merged the data from younger and older subjects and carried out the same correlations of PC with the above mentioned variables separately for the two allele types. These tests yielded a significant correlation between PC and Raven's matrices score in anyA9 carriers (r = 0.3486, p_(FDR)_ = 0.0072) but not in A10/A10 carriers (Fig 4). None of the other correlation coefficients reached significance after correction. However, this relation is largely associated with age-related declines in general cognitive abilities and the effect was eliminated after controlling for the effects of age (p > 0.05). As for the relation between PC and individual differences in excutive control function, interestingly individual differences in PC correlated positively with the accuracy of Stroop task performance in the incongruent color condition even after partialling out the effect of age in the anyA9 group (r = 0.2696, p_(FDR)_ = 0.0432), but not in the A10/A10 group (Fig 5).

## Discussion

Before discussing the results, the key findings are summarized here to provide an overview. At the brain-wise level, neither age nor genotype main effects were observed in the measures modularity (Q) and betweenness centrality (BC). In contrast, however, the measure PC, which quantifies the extent to which the network connectivity is evenly distributed across modules, yielded significant effects of age, genotype and their interactions. Specifically, the rsFC is less evenly distributed across modules (lower values of PC) in older than in younger adults in carriers of the *DAT1* anyA9 genotype, but not in A10 homozygotes. Furthermore, follow-up analyses revealed that the nodes contributing to the age and genotype effects with respect to PC mainly resided in the DMN network. None of the remaining six cortical networks using the Yeo et al. [50] parcellation–i.e., the visual (VIS), somatomotor (SOM), limbic (LM), dorsal attention (DOR), ventral attention/saliency (VEN), and frontoparietal (FPC) networks–exhibited age or genotype effects. Moreover, the values of PC correlated with individual differences in general cognitive ability and executive control function in *DAT1* anyA9 carriers, but not in A10 homozygotes. The functional relevance of PC in general cognitive ability is mainly shared with the age effect, whereas PC predicted individual differences in executive control above and beyond the effects of age.

The fact that we did not observe significant age differences in modularity (Q) is somewhat surprising, given previous results of either moderate negative correlation between age and Q in continuous adult developmental samples (e.g., [39]) or a significant age effect in age comparative samples (e.g., [21]). The discrepancies between our and prior studies might reflect that our older adult sample was relatively healthy, since previous studies included also older subjects with mild cognitive impairment and mild dementia [39]. Besides, differences in the parcellation schemes [21] used between studies may also contribute to the inconsistency. In line with previous results showing null relations between dementia status and the amount of nodes that fall within the shortest paths between other nodes within a network, we also did not observe effects of age or *DAT1* genotype on betweenness centrality (BC) [39].

To the best of our knowledge, this is the first study showing that adult age differences in rsFC could be moderated by individual differences in dopamine genotype. Depending on the ranges, values of PC may reflect different roles of the nodes in brain networks. Specifically, nodes with values of PC in the range (0.05 < PC < = 0.62) are known as peripheral nodes, which have most of their connections within rather than between modules; whereas nodes with values of PC in the range (0.62 < PC < = 0.80) or in the range (PC > 0.8), characterize connector nodes and kinless nodes, respectively. Connector and kinless nodes have more distributed connections across network modules [56]. The values of PC observed in our data are within the range of peripheral nodes. Findings from the correlational analyses suggest that individual differences in the extent of peripheral nodes’ connectivity positively predict individual differences in executive control function and general cognitive ability, albeit the latter effect is shared with the effect of age. These findings are in line with a recent study showing that PC as a metric of rsFC is predictive of individual differences in general intelligence [60].

A pattern of larger values of PC in older adults than in younger adults has been previously observed in a higher range of PC values for connector and kinless nodes. At these higher ranges, increasing values of PC indicate a tendency for uniformly distributed connections across modules. Aging-related increase of PC in the ranges of connector and kinless nodes were interpreted as an indication of age-related decrease in large-scale brain system segregation (e.g., [18]) that may underlie the established findings of aging-related cognitive dedifferentiation observed at the behavioral level [44, 61]. However, other prior aging studies which observed values of PC in the range of peripheral nodes, like our findings here, found age-related increases in values of PC either only in the visual and somatomotor networks but not in the DMN or FPC network [21], or only during cognitive tasks but not during resting state [62]. Furthermore, in general whether PC increases or decreases with age needs to be considered in light of the spatial scales of the networks. Similar to our findings, regarding regional integration in the DMN network, graph-theoretic analyses that consider multiple spatial scales show age-related decrease in PC [63].

Our findings extend the still limited literature on dopamine modulation of rsFC. Unlike findings from seed-based approaches [8, 27], in younger adults we did not observe a *DAT1* genotype effect on individual differences in internetwork connectivity as reflected in the PC of peripheral nodes. In part, this may be attributed to the fact that our observed effects are mainly in the DMN, whereas the prior finding based on dorsal caudate seeded rsFC mainly revealed effects in the attention network. The graph-theoretical approach, however, does not dependent on the selection of seed regions and is not affected by age or genotype differences in functional or structural integrities of the seed regions. Of particular interest is the genotype by age interaction, showing that correlated spontaneous brain activities are less distributed between network modules in older carriers of the *DAT1* anyA9 genotype than older A10 homozygotes. This result is, in part, in line with prior findings of positive correlations between dopamine transporter availability and regional rsFC between the midbrain and putamen in patients suffering from dopamine loss [26]. Furthermore, the fact that the genotype effect on the PC measure has been apparent in older but not in younger adults is also in line with several previous findings showing age-related magnification of genotype effects on cognitive functional outcomes [37, 64]. Nevertheless, further studies are needed to substantiate this age by genotype interaction, which suggests an age-related, DMN-specific decrease of intermodular communication in cortical networks in anyA9 carriers of the dopamine transporter gene.

This study is subject to the following limitations which need to be considered when interpreting the results. We used the cortical atlas by Yeo et al. [50] which was created by estimating intrinsic functional connectivity in a sample of young adults (18–35 years). Its parcellation might therefore not transfer well to the sample of older subjects in our study who might have different intrinsic functional coupling. Nevertheless, the parcellation scheme by Yeo et al. [50] was also used in prior aging and lifespan studies [15, 18, 19]. Furthermore, the aforementioned atlas does not contain subcortical areas which prevented us from exploring whether regions that receive substantial dopaminergic input, such as striatum or hippocampus, also show *DAT1* genotype related differences in functional network characteristics. In particular, in light of evidence revealing higher dopamine transporter density in midbrain regions than in the cortex [65–67], future studies would need to use finely parcellated maps of the human striatum [68] to investigate potential interactive effects of *DAT1* genotype and aging on graph theoretical metrics of intrinsic functional connectivity in the striatal subnetwork. In terms of better controlling for potential confounds, in light of a recent exploratory finding of systolic blood pressure being negatively associated with dopamine transporter variability in older adults [69], future studies need to also assess indicators of vascular health and intake of medication for controlling blood pressure, particularly in older samples. Finally, for investigating both age and genotype related effects on brain network topology our study sample is rather small, potentially underpowering our statistical analyses. Our lack of findings of age differences regarding modularity and the absence of significant correlations between our four age and genotype groups regarding PC might be due to low statistical power.

### Conclusion and outlook

We found age by genotype interaction effects in resting-state fMRI networks that indicate a lower degree of intermodular communication in older adults compared to younger adults who are anyA9 allele carriers of the dopamine transporter gene *DAT1*, while this effect was absent in A10 homozygotes. Furthermore, the anyA9 genotype was positively correlated with executive control and general cognitive ability, although the latter effect can be partially explained by aging. Taken together, these findings suggest a modulating effect of dopamine on functional brain networks at rest and a possible impact of said network characteristics on cognitive functioning.

Future studies should expand on these findings and investigate how age and other dopamine genotypes may interact in affecting functional connectivity patterns during performance on tasks that depend on dopamine functioning in larger samples. Such an approach would more directly relate connectivity to cognition than correlating graph-theoretic measures of resting state networks with behavior in offline tasks. Further studies are also needed to examine whether pharmacological interventions (e.g. the administration of levodopa) are able to alter functional networks in different age groups. As some studies point towards a link between brain network topology and cognitive performance [70, 71], it would be an interesting question to test if potential pharmaco-induced improvements in task performance are reflected in changes of interactions between brain regions in older adults. Another interesting avenue of research would be to explore when, during the course of the later lifespan, brain network changes become apparent. Via longitudinal studies potential interrelationships between the emergence of alterations in functional connectivity and changes in cognitive performances could be investigated.

## Supporting information

S1 Fig No age and genotype main effects in modularity (Q). P values are plotted as a function of K while the horizontal black line indicates the FDR corrected p = 0.05. Abbreviations: FDR, false discovery rate; K, node-degree; OA, old adults; YA, young adults.
(TIFF)

S2 Fig No age by genotype interaction regarding modularity (Q). P values are plotted as a function of K, while the horizontal black line indicates the FDR corrected p = 0.05. Abbreviations: FDR, false discovery rate; K, node-degree; OA, old adults; YA, young adults.
(TIFF)

S3 Fig No age and genotype main effects in betweenness centrality (BC). P values are plotted as a function of K while the horizontal black line indicates the FDR corrected p = 0.05. Abbreviations: FDR, false discovery rate; K, node-degree; OA, old adults; YA, young adults.
(TIFF)

S4 Fig No age by genotype interaction regarding betweenness centrality (BC). P values are plotted as a function of K, while the horizontal black line indicates the FDR corrected p = 0.05. Abbreviations: FDR, false discovery rate; K, node-degree; OA, old adults; YA, young adults.
(TIFF)

S5 Fig No age or genotype main effects in participation coefficient (PC). P values are plotted as a function of K while the horizontal black line indicates the FDR corrected p = 0.05. Abbreviations: FDR, false discovery rate; K, node-degree; OA, old adults; YA, young adults.
(TIFF)

S6 Fig No age by genotype interaction regarding participation coefficient (PC) in 6 YEO sub-networks. P values are plotted as a function of K, while the horizontal black line indicates the FDR corrected p = 0.05. Abbreviations: FDR, false discovery rate; K, node-degree; OA, old adults; YA, young adults. VIS: visual network; DOR: dorsal attention network; LM: limbic network; N, number of nodes within subnetwork; SOM: somatomotor network; VEN: ventral attention network; FPC; frontoparietal network.
(TIFF)
