# Supplementary figures and images for: Interactive effects of dopamine transporter genotype and aging on resting-state functional networks
Source: PLoS One. 2019 May 8;14(5):e0215849. doi: 10.1371/journal.pone.0215849 (PMC6505745; doi:10.1371/journal.pone.0215849)

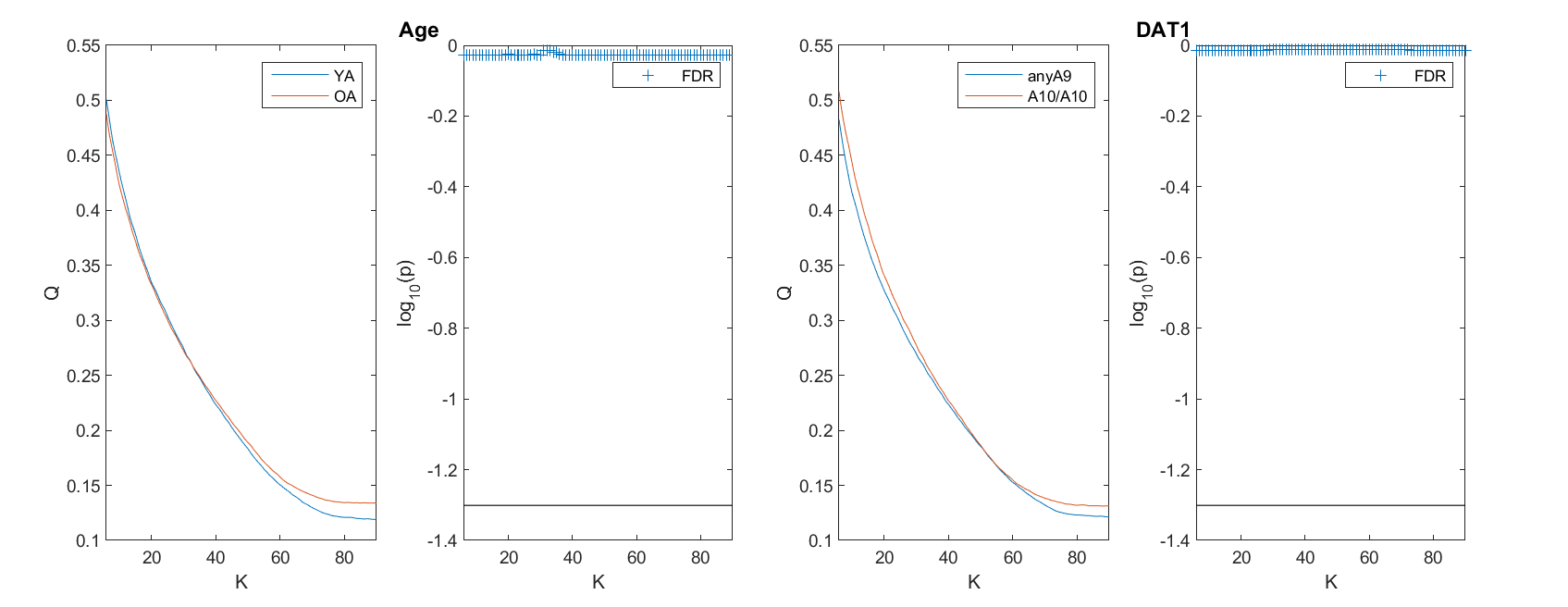

Supplement: S1 Fig — P values are plotted as a function of K while the horizontal black line indicates the FDR corrected p = 0.05. Abbreviations: FDR, false discovery rate; K, node-degree; OA, old adults; YA, young adults. (TIFF) [file pone.0215849.s001.tiff]

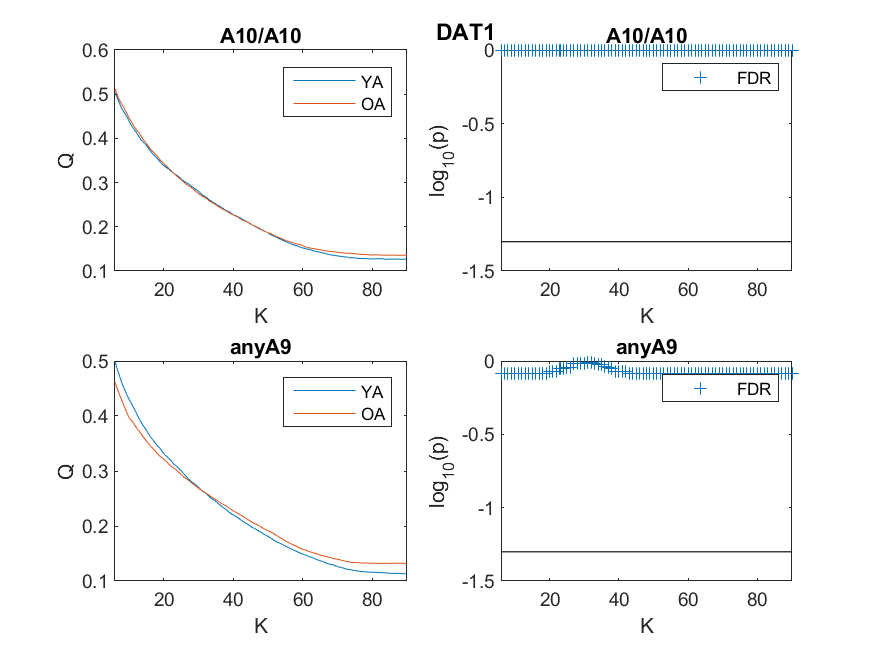

Supplement: S2 Fig — P values are plotted as a function of K, while the horizontal black line indicates the FDR corrected p = 0.05. Abbreviations: FDR, false discovery rate; K, node-degree; OA, old adults; YA, young adults. (TIFF) [file pone.0215849.s002.tiff]

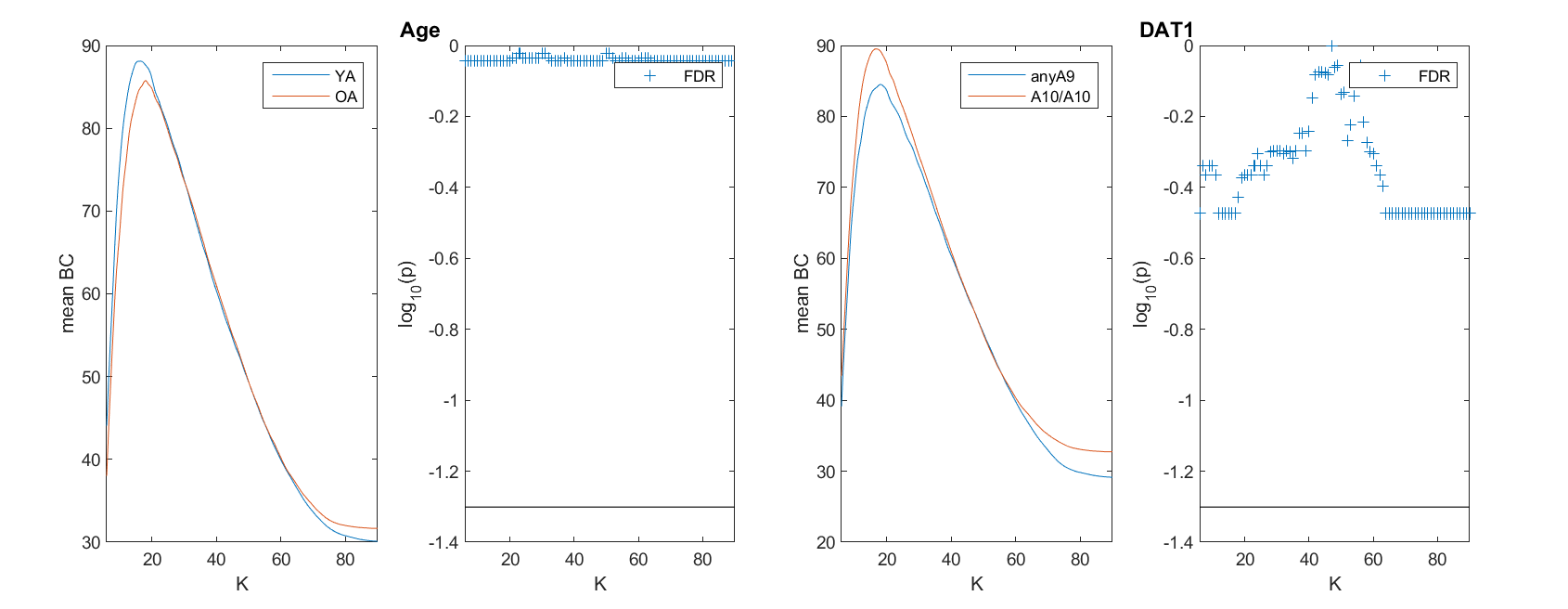

Supplement: S3 Fig — P values are plotted as a function of K while the horizontal black line indicates the FDR corrected p = 0.05. Abbreviations: FDR, false discovery rate; K, node-degree; OA, old adults; YA, young adults. (TIFF) [file pone.0215849.s003.tiff]

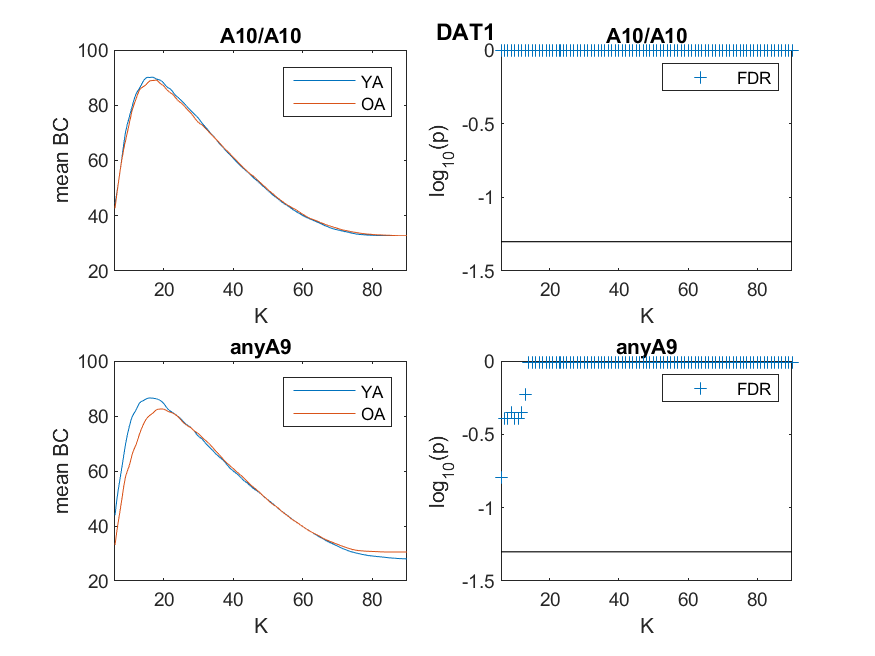

Supplement: S4 Fig — P values are plotted as a function of K, while the horizontal black line indicates the FDR corrected p = 0.05. Abbreviations: FDR, false discovery rate; K, node-degree; OA, old adults; YA, young adults. (TIFF) [file pone.0215849.s004.tiff]

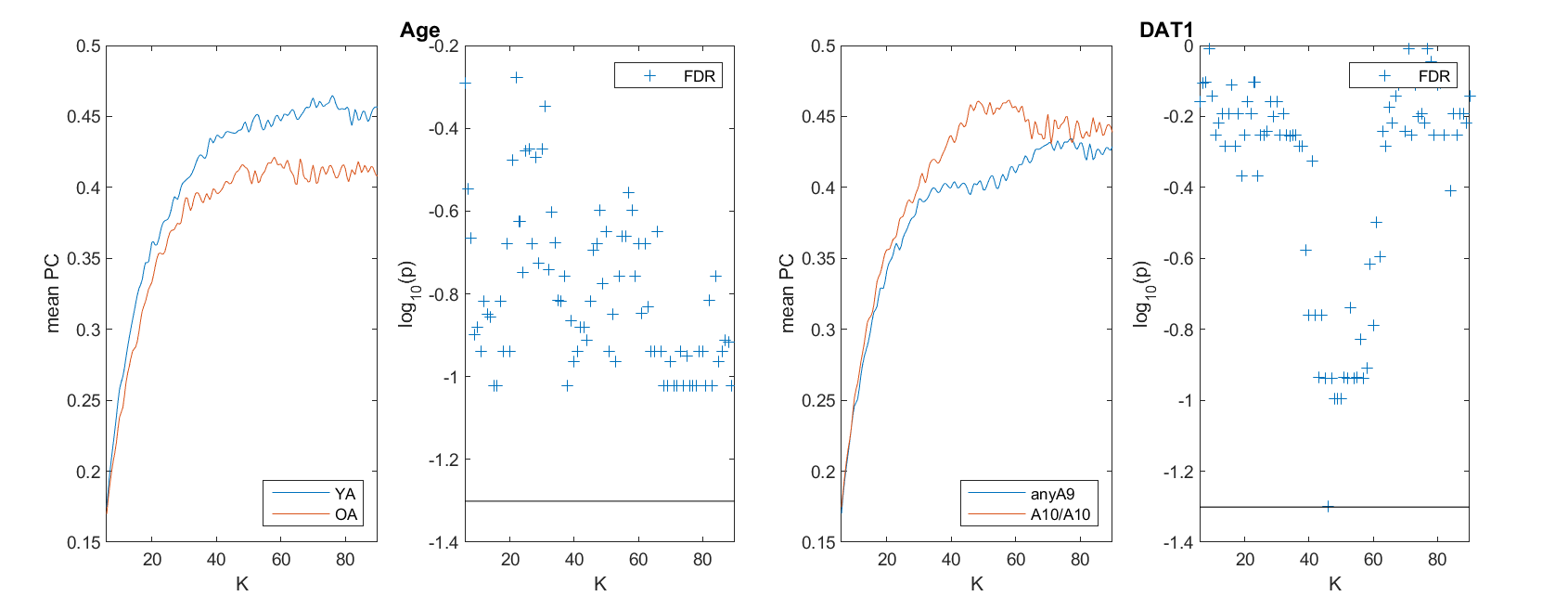

Supplement: S5 Fig — P values are plotted as a function of K while the horizontal black line indicates the FDR corrected p = 0.05. Abbreviations: FDR, false discovery rate; K, node-degree; OA, old adults; YA, young adults. (TIFF) [file pone.0215849.s005.tiff]

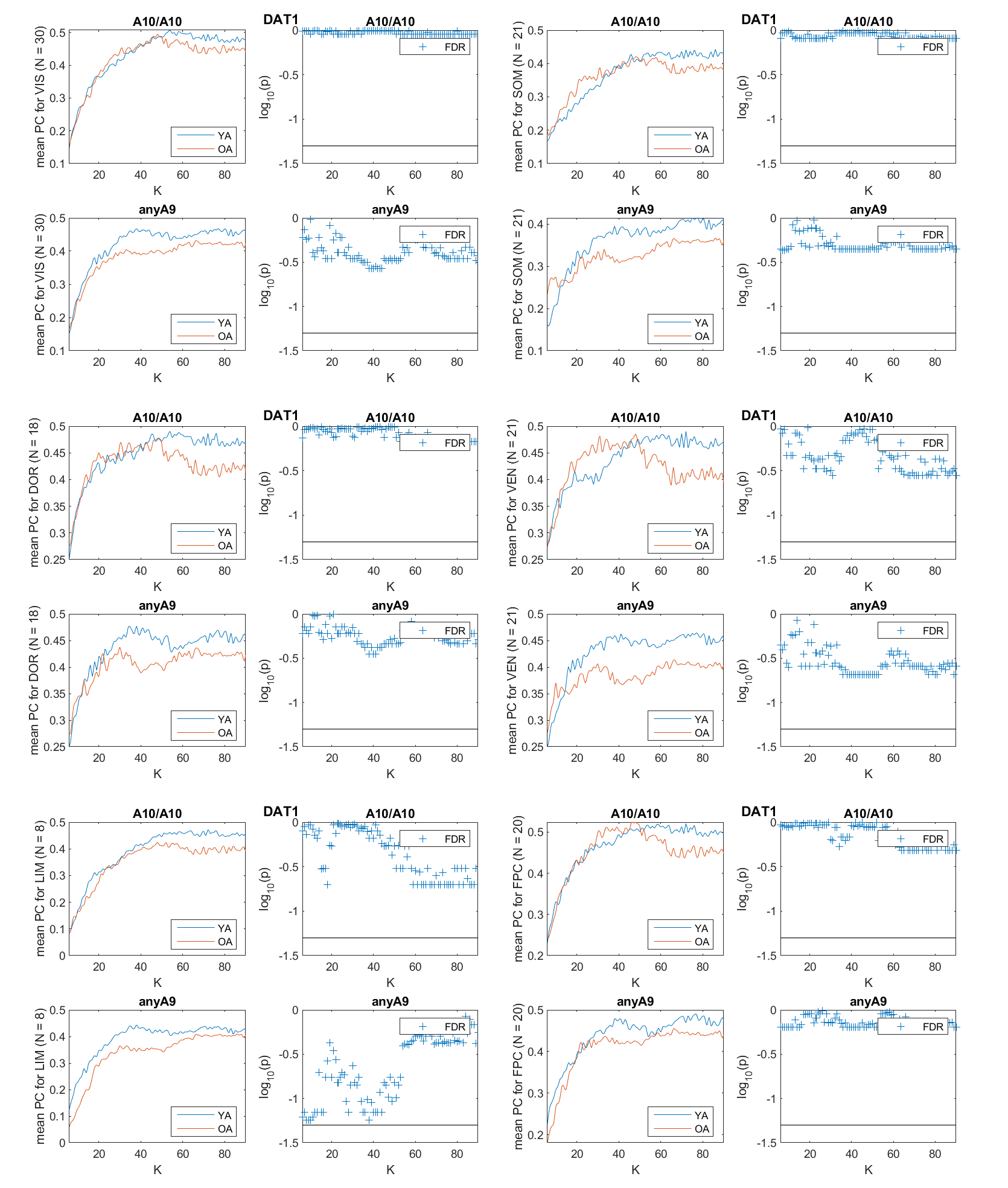

Supplement: S6 Fig — P values are plotted as a function of K, while the horizontal black line indicates the FDR corrected p = 0.05. Abbreviations: FDR, false discovery rate; K, node-degree; OA, old adults; YA, young adults. VIS: visual network; DOR: dorsal attention network; LM: limbic network; N, number of nodes within subnetwork; SOM: somatomotor network; VEN: ventral attention network; FPC; frontoparietal network. (TIFF) [file pone.0215849.s006.tiff]
